# Normal pre-attentive and impaired attentive processing of lexical tones in Cantonese-speaking congenital amusics

**DOI:** 10.1038/s41598-018-26368-7

**Published:** 2018-05-30

**Authors:** Caicai Zhang, Jing Shao

**Affiliations:** 1 Department of Chinese and Bilingual Studies, The Hong Kong Polytechnic University, Hong Kong SAR, China; 2 Shenzhen Institutes of Advanced Technology, Chinese Academy of Sciences, Shenzhen, 518055 China

## Abstract

The neural underpinnings of congenital amusia, an innate neurogenetic disorder of musical pitch processing, are not well understood. Previous studies suggest that amusia primarily impairs attentive processing (P300) of small pitch deviations in music, leaving pre-attentive pitch processing (mismatch negativity or MMN) more or less intact. However, it remains unknown whether the same neuro-dynamic mechanism of deficiency underlies pitch processing in speech, where amusics also often show impairment behaviorally. The current study examined how lexical tones are processed in pre-attentive (MMN) and attentive (P300) conditions in 24 Cantonese-speaking amusics and 24 matched controls. At the pre-attentive level, Cantonese-speaking amusics exhibited normal MMN responses to lexical tone changes, even for tone pairs with small pitch differences (mid level vs. low level tone; high rising vs. low rising tone). However, at the attentive level, amusics exhibited reduced P3a amplitude for all tone pairs, and further reduced P3b amplitude for tone pairs with small pitch differences. These results suggest that the amusic brain detects tone changes normally pre-attentively, but shows impairment in consciously detecting the same tone differences. Consistent with previous findings in nonspeech pitch processing, this finding provides support for a domain-general neuro-dynamic mechanism of deficient attentive pitch processing in amusia.

## Introduction

Congenital amusia (amusia hereafter) is a lifelong neurogenetic disorder primarily influencing musical pitch processing in about 1.5–4% of the population^1–11^. While some earlier studies claim that amusia is a domain-specific pitch deficit^2,12–14^, recent studies found that this disorder extends to pitch processing in speech, giving rise to poor performance in speech intonation processing^15–19^, emotion prosody processing^20^, and lexical tone processing^5,11,21–26^, especially if the pitch differences are small. Based on these results, it has been suggested that amusia is a domain-general pitch deficit, affecting refined pitch processing in music as well as speech^27^.

Although the behavioral deficits of amusia in music and speech perception are extensively studied, relatively little is known about the neuro-dynamic mechanism of the pitch deficiency in amusia at different processing levels along the auditory pathway. One line of evidence indicates that pre-attentive pitch processing may be normal in the amusic brain, and that the deficit primarily lies in the conscious detection of pitch differences^8,28–30^. Consistent with this claim, an event-related potentials (ERPs) study showed that the brain activities of amusics can track a musical note mistuned by a quarter-tone, whereas they have difficulties behaviorally detecting the mistuned note^29^. Providing further evidence for this claim, another ERPs study reported that the mismatch negativity (MMN), an early automatic cortical response to auditory changes in a stream of repetitive auditory stimuli without subjects’ focal attention^31,32^, was normal at detecting pitch deviations as small as 25 cents in the amusic brain^30^. Instead, when the amusics were asked to pay attention to the auditory stimuli and actively detect such small pitch deviations, they showed no P3b response, unlike the controls^30^. Normal MMN response in the passive listening condition but absence of P3b response in the active condition suggests that pitch processing may be more or less normal at a pre-attentive level and impaired at a later attentive level in the amusic brain.

The other line of evidence indicates that pre-attentive processing of pitch differences may already be impaired in some amusics^33^. It has been found that pre-attentive auditory processing of lexical tones, as indexed by the MMN, was abnormal in a subgroup of Mandarin-speaking amusics with severe tone perception impairment behaviorally, namely tone agnosics, but not in another subgroup of Mandarin-speaking amusics without severe tone perception impairment behaviorally, namely pure amusics^33^. The tone agnosia subgroup showed reduced MMN responses to lexical tone changes compared to pure amusics as well as musically intact controls, but their MMN responses to consonant changes were normal. Furthermore, neural deficits in the auditory cortex of the amusic brain have been reported in previous studies^9,10,34^. As the primary neural source of MMN is located in auditory cortices^35,36^, it is possible that deficient neural processing in auditory cortices might affect MMN activities.

While the aforementioned findings are informative, relatively little is known about the neuro-dynamic mechanism of the pitch-processing deficit in speech at different processing levels. As no studies have systematically compared pitch processing in speech at pre-attentive and attentive levels in amusia, it remains unknown whether the amusics’ pitch-processing deficiency in speech is primarily manifested at attentive levels, or whether pre-attentive pitch processing in speech is already impaired.

In order to test the two hypotheses above, the current study uses lexical tones, namely pitch differences systematically distinguishing lexical meanings in tonal languages^37,38^, to probe the neuro-dynamic mechanism of pitch processing in speech in amusia. There are two reasons for focusing on lexical tones. Firstly, pitch differences that distinguish lexical tones are relatively small (e.g., two semitones) compared to pitch differences that index speech intonation patterns (e.g., statement/question). Secondly, previous studies on lexical tone perception have consistently reported behavioral impairment in amusics^5,11,22–26^, whereas results on speech intonation perception deficits in amusia were less conclusive^13,15–19^. It has been found that Mandarin-speaking amusics performed generally worse than controls in the identification and discrimination of Mandarin tones. Furthermore, there were subgroup differences among amusics in terms of the severity of tone perception impairment. Those amusics who performed 3 SDs below the mean accuracy of controls were referred to as tone agnosics and the rest was referred to as pure amusics^5^. Amusic individuals whose mother tongue is Cantonese, a highly complex tonal language^39^, are also found to perform less accurately than controls in the identification and discrimination of native tones^21,23^. The impairment of Cantonese-speaking amusics in tone discrimination may be less severe compared to that in tone identification, and is primarily manifested in the discrimination of tone pairs with small pitch differences^23^. For the above reasons, lexical tones are well suited for examining the neuro-dynamic mechanism of pitch processing in speech in amusia.

**Figure 1:**
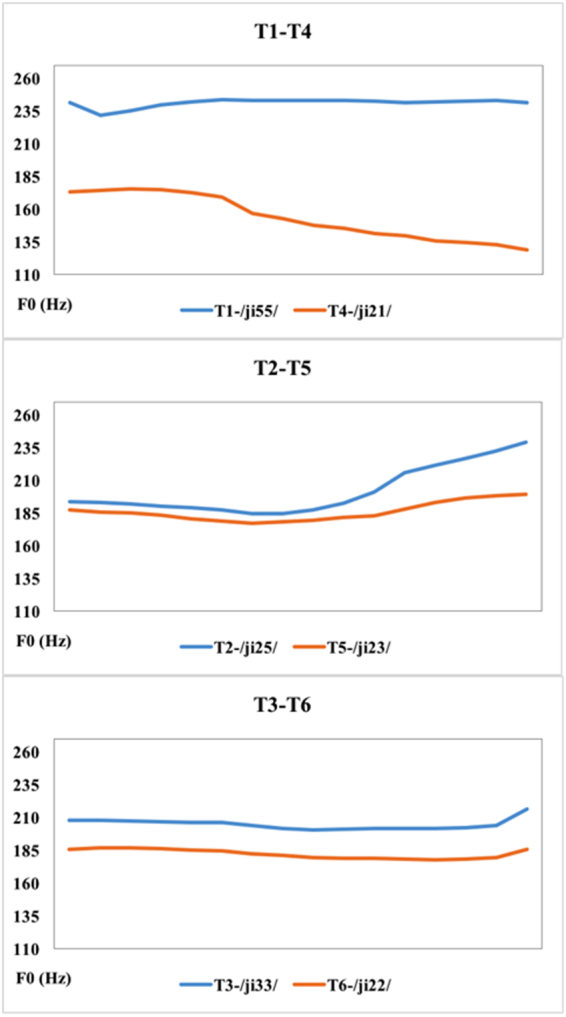
F0 curve of the three tone pairs (T1-T4, high level-extra low level/low falling tone; T3-T6, mid level-low level tone; T2-T5, high rising-low rising tone) used as stimuli in the experiments.

To this end, the current study examined the ERP correlates of lexical tone processing with and without attention in 24 Cantonese-speaking amusics and 24 matched controls. Neural activities during the processing of Cantonese tone pairs with small pitch differences (mid level-low level tone, or T3-T6; high rising-low rising tone, or T2-T5) and large pitch differences (high level-low falling tone, or T1-T4) (see Fig. 1) were compared in passive (MMN) and active (P300) listening conditions. If the same neuro-dynamic mechanism previously reported on nonspeech pitch processing^8,28–30^ underlies lexical tone processing, Cantonese-speaking amusics are expected to show normal pre-attentive processing of lexical tones, even for tone pairs with small pitch differences, but impaired attentive processing of lexical tones. Alternatively, it is possible that the neuro-dynamic mechanism of lexical tone processing is different from what is previously reported on nonspeech pitch processing, and starts to exhibit impairment at a preattentive level. As mentioned above, pre-attentive processing of lexical tone differences is found to be impaired in Mandarin speakers with tone agnosia^33^. In light of this finding, it is possible that Cantonese-speaking amusics may show impaired neural processing of pitch from the pre-attentive level, which persists into the attentive level.

## Results

### Passive condition

**Figure 2:**
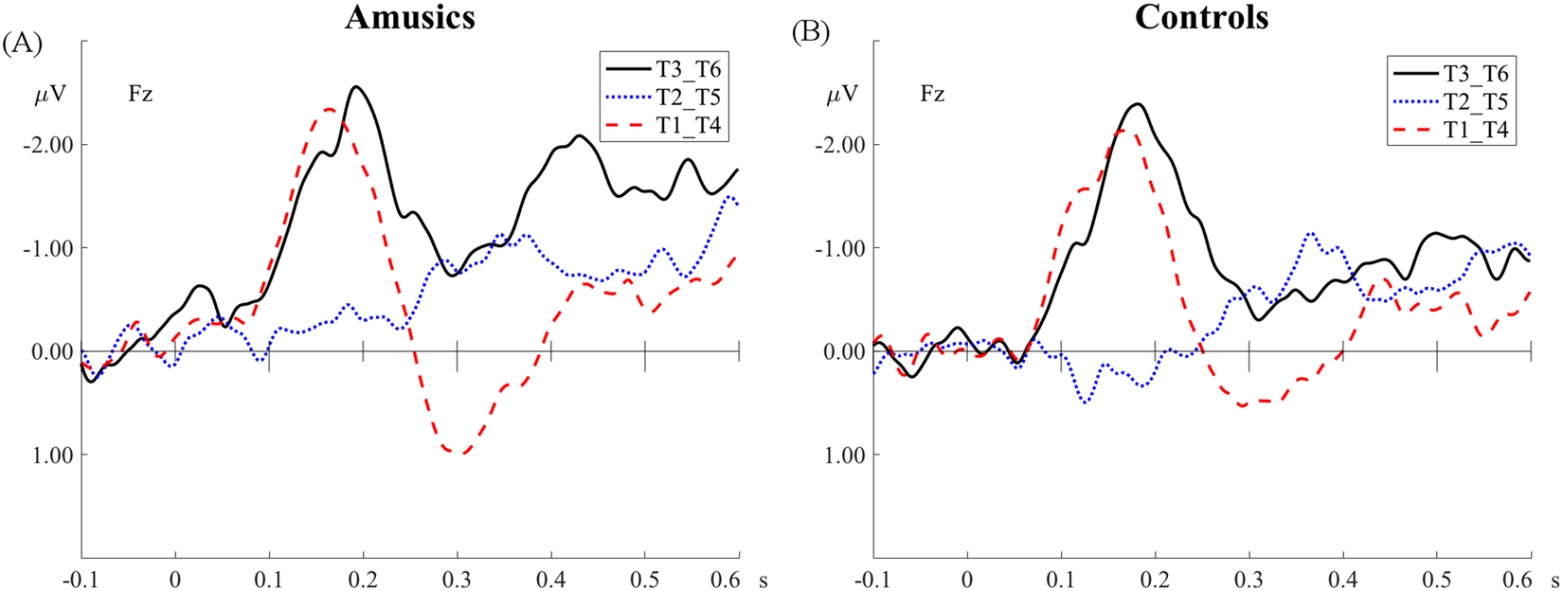
Difference waveforms of the three tone pairs in the passive condition. (**A**) The amusic group. (**B**) The control group.

**Figure 3:**
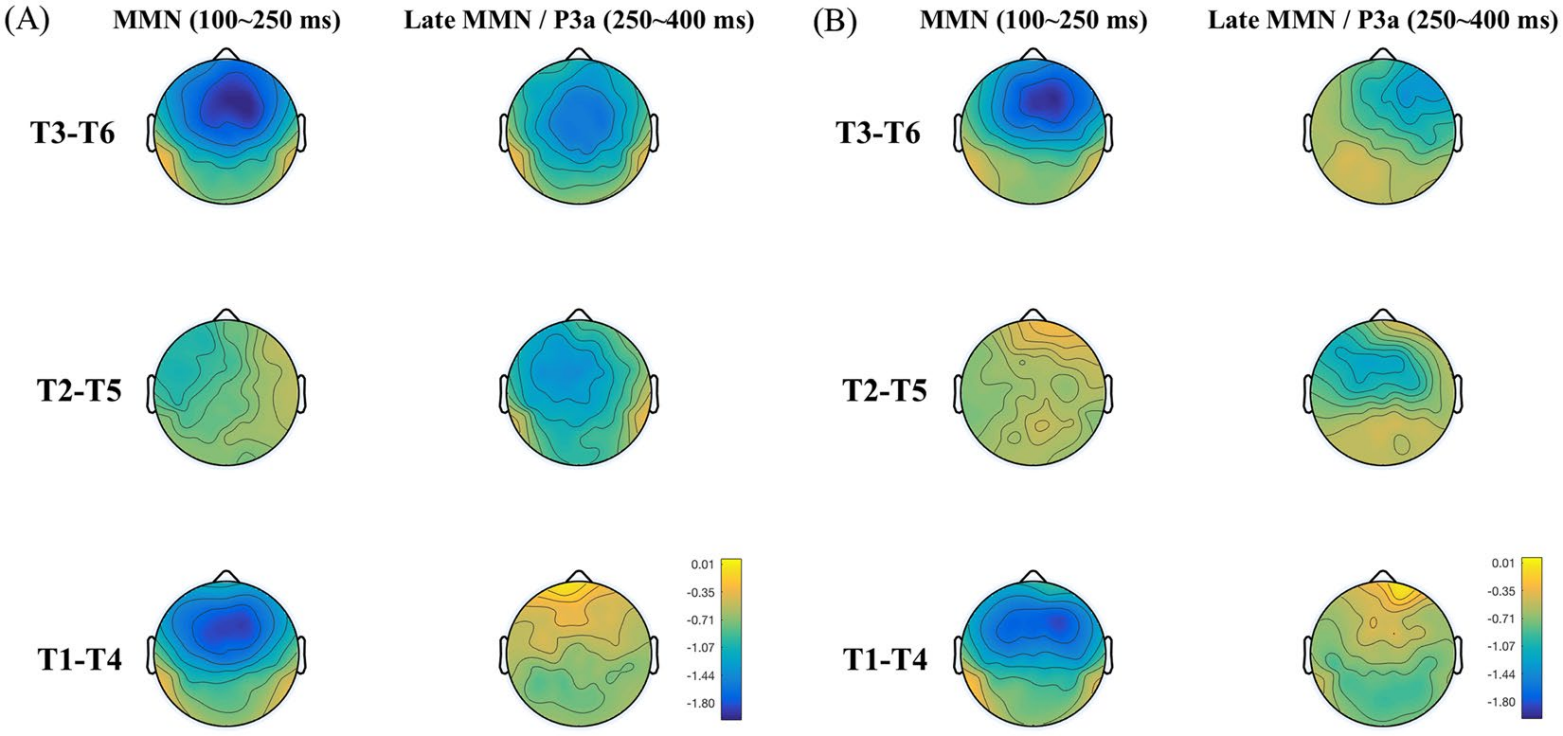
Topographic distributions of the MMN (100–250 ms) and late MMN/P3a (250–400 ms) of the three tone pairs. (**A**) The amusic group. (**B**) The control group.

Figures 2 and 3 show the MMN and its topographic distribution in the amusic and control group. As can be seen in Fig. 2, for both amusics and controls the MMN peaked in the time-window of 100–250 ms for tone pairs T1-T4 and T3-T6, but it appeared to be delayed for the T2-T5 pair, occurring in a later time-window of approximately 250–400 ms. This delay is expected because the pitch deviance between T2 and T5 occurred later than the other two tone pairs (see Fig. 1). Also in this late time-window (250–400 ms), the T1-T4 pair appeared to elicit a P3a following its MMN, which may be due to involuntary attention switch to the acoustically salient tonal change in this pair^40^. Analyses were conducted on these two time windows: MMN (100–250 ms) and late MMN/P3a (250–400 ms). The peak latency and mean amplitude of MMN, late MMN and P3a were analyzed.

**Figure 4:**
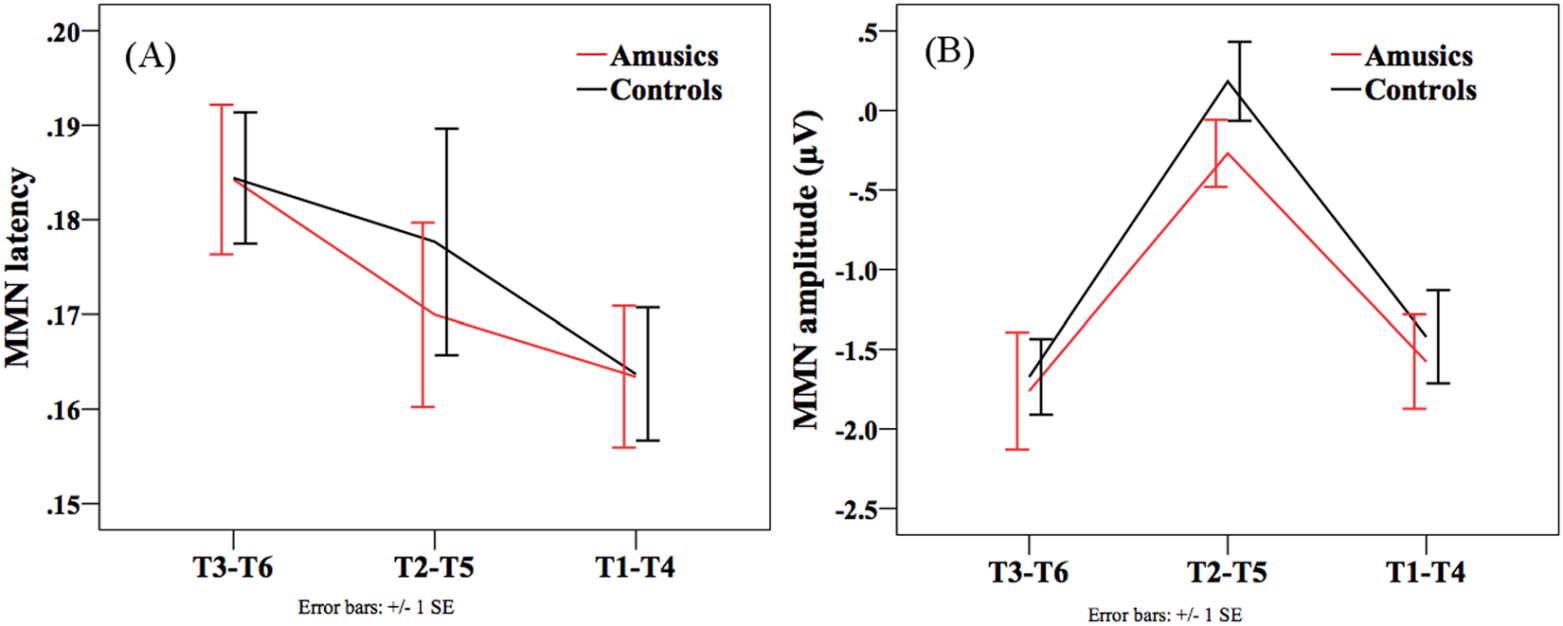
MMN results (100–250 ms) for the three tone pairs and two groups. (**A**) MMN peak latency. (**B**) MMN mean amplitude.

For the MMN peak latency, *group* (amusics vs. controls) × *tone pair* (T1-T4 vs. T3-T6 vs. T2-T5) ANOVA only revealed a main effect of *tone pair* (*F*(2, 92) = 3.155, *p* = 0.047). Pairwise comparisons showed that the T1-T4 pair peaked significantly earlier than the T3-T6 pair (*p* = 0.010). For the MMN amplitude, again, there was only a significant main effect of *tone pair* (*F*(2, 92) = 23.34, *p* < 0.001). The T2-T5 pair elicited significantly smaller MMN amplitude than the other two pairs in the 100–250 ms time-window (*p*s < 0.001). No effects of *group* or *group* × *tone pair* were significant. These results reflected the modulation effect of acoustic distance of different tone pairs on the MMN latency and amplitude, such that the T2-T5 pair with small and late pitch differences elicited a reduced and delayed MMN, while the T1-T4 pair with large pitch differences elicited the earliest-peaking MMN. Figure 4 shows the MMN latency and amplitude.

In the late time-window (250–400 ms), independent-samples t-tests were conducted to analyze whether there were group differences on the late MMN for the T2-T5 pair, and on the P3a for the T1-T4 pair, respectively. Because the late MMN was elicited in the T2-T5 pair and the P3a was elicited in the T1-T4 pair, t-tests focusing on these tone pairs were conducted respectively, while the other tone pairs were not included in the analyses. For the T2-T5 pair, no significant group difference was found for either the late MMN latency (*t*(46) = 0.170, *p* = 0.866) or amplitude (*t*(46) = −0.441, *p* = 0.661). For the T1-T4 pair, again, the group difference was not significant for either the P3a latency (*t*(46) = 1.026, *p* = 0.310) or amplitude (*t*(46) = 0.300, *p* = 0.766).

### Active condition

**Figure 5:**
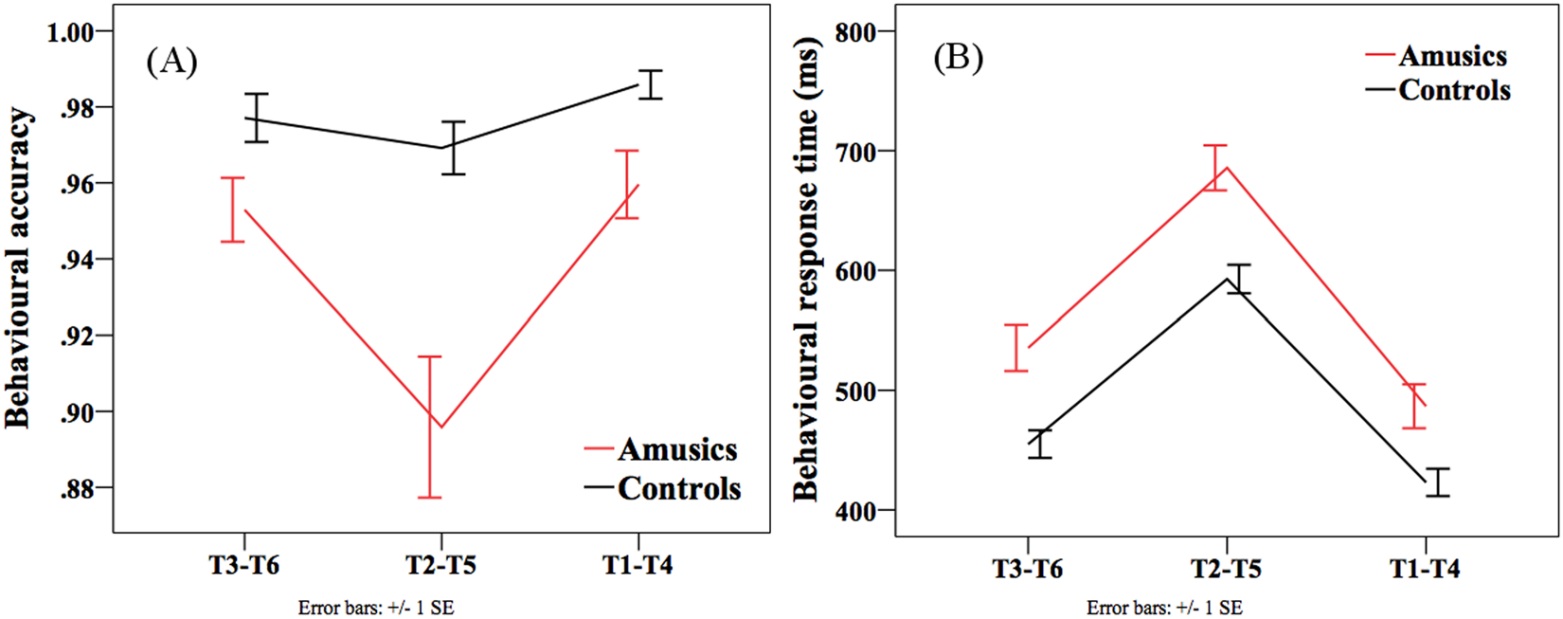
Behavioral performance in the active condition. (**A**) Accuracy of the two groups of subjects in detecting deviants of the three tone pairs. (**B**) Reaction time of the two groups of subjects in detecting deviants of the three tone pairs.

**Figure 6:**
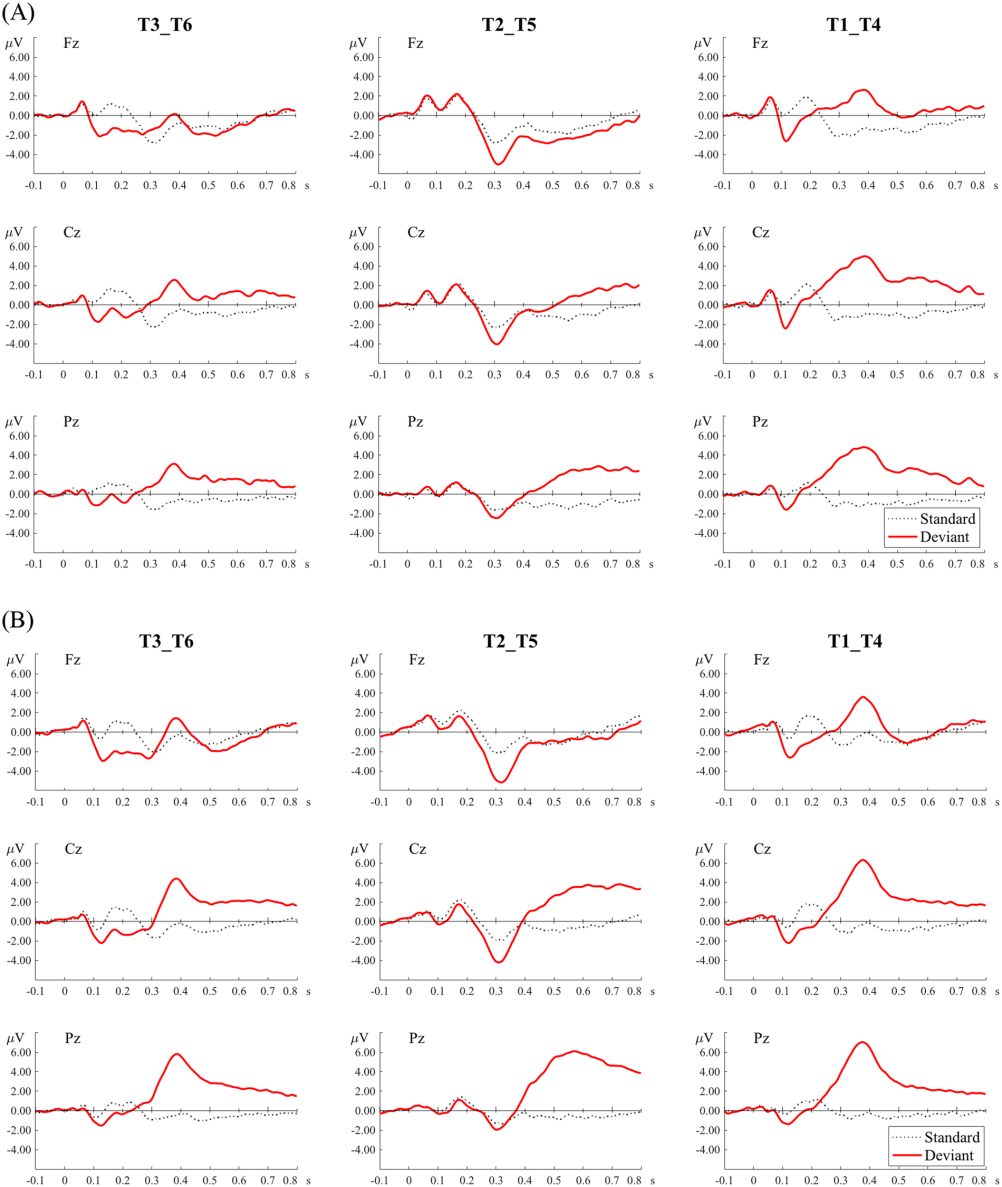
Grand average ERP waveforms of the standards and deviants of the three tone pairs in the active condition. (**A**) The amusic group. (**B**) The control group.

**Figure 7:**
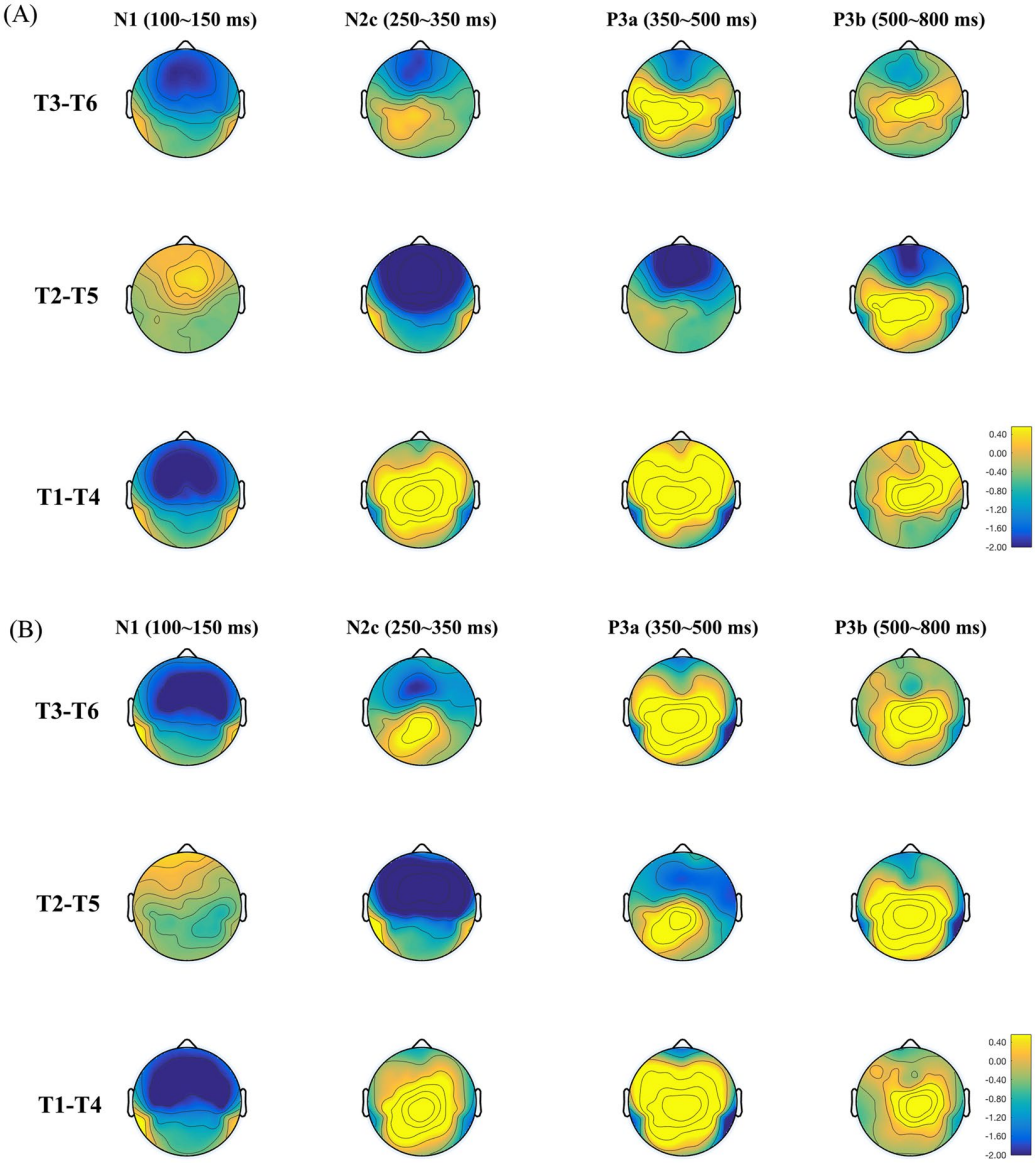
Topographic distributions of the N1 (100–150 ms), N2b/c (250–350 ms), P3a (350–500 ms), and P3b (500–800 ms) of the three tone pairs. (**A**) The amusic group. (**B**) The control group.

**Figure 8:**
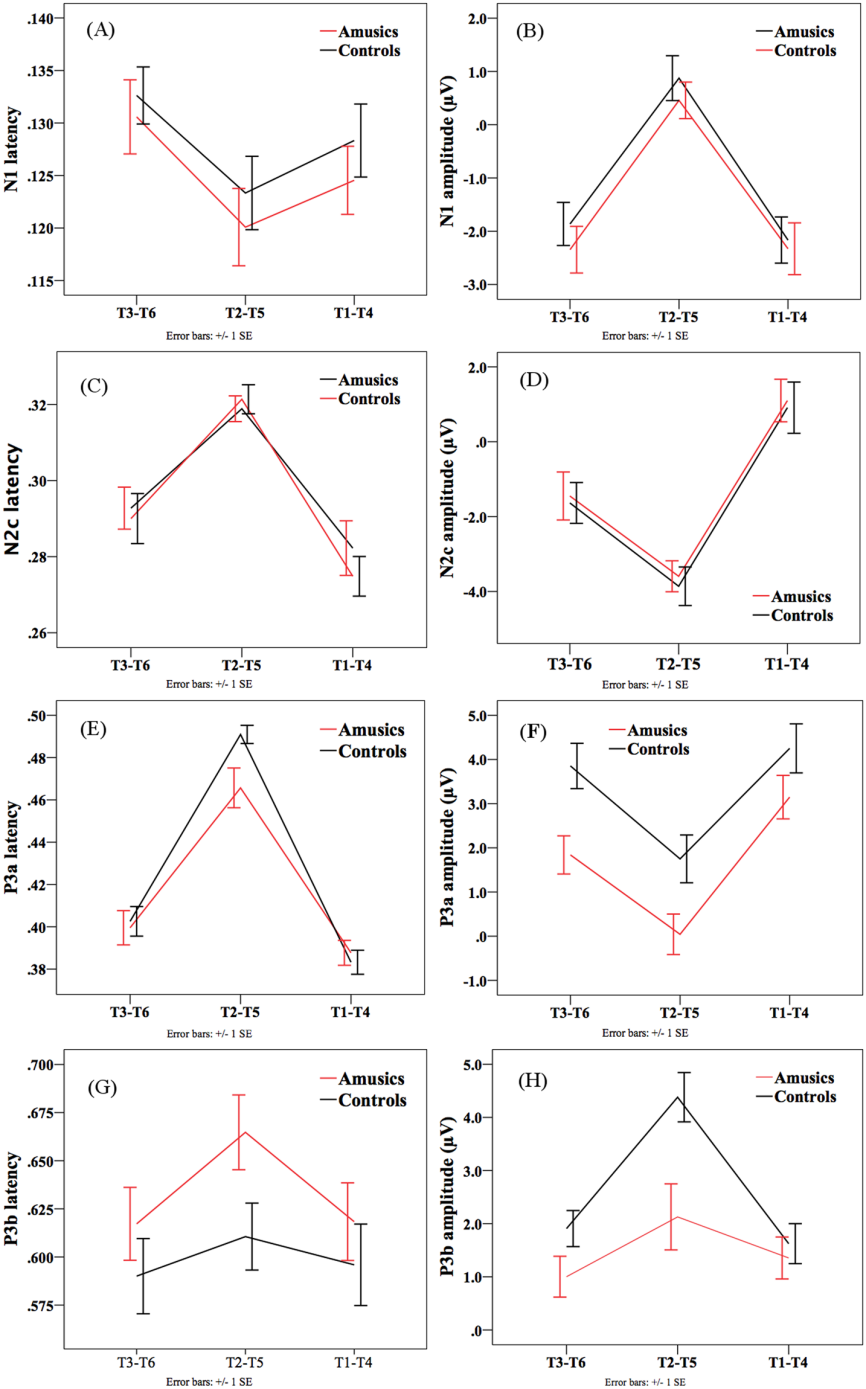
ERP results in the active condition. (**A**) N1 (100–150 ms) peak latency. (**B**) N1 (100–150 ms) mean amplitude. (**C**) N2b/c (250–350 ms) peak latency. (**D**) N2b/c (250–350 ms) mean amplitude. (**E**) P3a (350–500 ms) peak latency. (**F**) P3a (350–500 ms) mean amplitude. (**G**) P3b (500–800 ms) peak latency. (**H**) P3b (500–800 ms) mean amplitude.

Figure 5 displays the accuracy and reaction time (RT) of the two groups in detecting tonal deviants in the active condition. Figures 6 and 7 shows the ERP waveforms of the three tone pairs and their topographic distributions. Analyses were conducted on the following four time-windows: N1 (100–150 ms), N2b/c (250–350 ms), P3a (350–500 ms) and P3b (500–800 ms). The peak latency and mean amplitude of these four ERP components are displayed in Fig. 8.

#### Behavioral results

For the accuracy, *group* × *tone pair* ANOVA found significant main effects of *group* (*F*(1, 46) = 12.911, *p* < 0.001), *tone pair* (*F*(1.278, 58.776) = 18.217, *p* < 0.001), and a significant two-way interaction (*F*(1.278, 58.776) = 7.731, *p* = 0.004). Independent-samples t-tests revealed that amusics performed significantly less accurately than controls on all three pairs (*p*s < 0.05), but the impairment of amusics was most pronounced on the T2-T5 pair (amusics = 89.6%; controls = 96.9%), compared with the T1-T4 (amusics = 96%; controls = 98.6%) and T3-T6 pairs (amusics = 95.3%; controls = 97.7%). Within the amusic group, there was a significant difference in the accuracy of the three tone pairs (*F*(2, 69) = 7.460, *p* = 0.001), in that the accuracy on the T2-T5 pair was significantly lower than that on T1-T4 and T3-T6 pairs (*p*s < 0.05). Within the control group, no significant effects were found. The results suggested that amusics were especially impaired in the detection of tonal changes in the T2-T5 pair with small pitch differences.

For the RT, there was a significant main effect of *group* (*F*(1, 46) = 14.735, *p* < 0.001), where amusics showed significantly overall longer RT than controls in detecting tonal changes. The main effect of *tone pair* was also significant (*F*(1.278, 72.694) = 373.126, *p* < 0.001). Pairwise comparisons showed that the RT of the T1-T4 pair was significantly shorter than that of T2-T5 and T3-T6 pairs (*p*s < 0.001), while the T3-T6 pair also elicited a shorter RT than the T2-T5 pair (*p* < 0.001). This result reflects that the speed of detecting tonal changes is closely tied to the magnitude of pitch differences of the three tone pairs. The two-way interaction was not significant.

#### N1

For the N1, there was only a significant main effect of *tone pair* for its latency (*F*(2, 92) = 6.876, *p* = 0.002) and amplitude (*F*(2, 92) = 87.742, *p* < 0.001). Pairwise comparisons revealed that the T2-T5 pair peaked significantly earlier than the T3-T6 pair (*p* = 0.005). The T2-T5 pair also elicited reduced (less negative) N1 amplitude than the other two pairs (*p*s < 0.05). No other effects were significant. These results generally reflected reduced auditory processing of tonal changes in the T2-T5 pair with small pitch differences.

#### N2b/c

For the N2b/c, again, there was only a significant main effect of *tone pair* on its latency (*F*(2, 92) = 39.52, *p* < 0.001), and amplitude (*F*(2, 92) = 54.5, *p* < 0.001). In terms of the latency, the T2-T5 pair peaked significantly later than the other two pairs (*p*s = 0.005), and the T3-T6 pair peaked later than the T1-T4 pair (*p* = 0.014). In terms of the amplitude, the T2-T5 pair elicited significantly larger (more negative) amplitude than the other two pairs (*p*s < 0.001), and the T3-T6 pair elicited larger amplitude than the T1-T4 pair (*p* < 0.001). These results may suggest that more attention resources were directed to detecting tonal changes in the T2-T5 pair with small pitch differences, followed by the T3-T6 pair, and finally the T1-T4 pair.

#### P3a

For the P3a latency, there was only a significant main effect of *tone pair* (*F*(1.615, 74.307) = 106.813, *p* < 0.001). The T2-T5 pair peaked significantly later than the T3-T6 pair (*p* < 0.001), which peaked later than the T1-T4 pair (*p* = 0.009).

For the P3a amplitude, there were significant main effects of *group* (*F*(1, 46) = 6.966, *p* = 0.011) and *tone pair* (*F*(1.612, 74.155) = 42.333, *p* < 0.001). The amusic group demonstrated significantly smaller P3a amplitude than the control group (1.677 µV vs. 3.285 µV). Among the three tone pairs, the T1-T4 pair elicited significantly larger P3a amplitude than the T3-T6 pair (*p* = 0.003), which elicited larger amplitude than the T2-T5 pair (*p* < 0.001). The two-way interaction was not significant.

#### P3b

For the P3b latency, no effects were significant.

For the P3b amplitude, there were significant main effects of *group* (*F*(1, 46) = 5.656, *p* = 0.022), *tone pair* (*F*(1.632, 75.064) = 17.976, *p* < 0.001), and a significant two-way interaction (*F*(1.632, 75.064) = 4.352, *p* = 0.016). Post-hoc analyses revealed that the amusic group exhibited reduced P3b amplitude than the control group for the T2-T5 pair (2.128 µV vs. 4.380 µV; *t*(46) = −2.902, *p* = 0.006); the group difference was marginally significant for the T3-T6 pair, where the amusic group again displayed reduced P3b amplitude (1.002 µV vs. 1.908 µV; *t*(46) = −1.767, *p* = 0.084); but the group difference was not significant for the T1-T4 pair (1.355 µV vs. 1.624 µV). Within the amusic group, the P3b amplitude was not significantly different among the three tone pairs (*F*(2, 69) = 1.440, *p* = 0.244). Within the control group, there was a difference among the three tone pairs (*F*(2, 69) = 14.571, *p* < 0.001), where the T2-T5 pair elicited significantly larger amplitude than T1-T4 and T3-T6 pairs (*p*s < 0.001).

#### Regression analyses

A series of regression analyses were carried out to examine to what extent the subjects’ tone change detection performance (accuracy and RT) and its underlying P300 activities can be explained by their musical ability. The predictors were the subjects’ accuracy in the three musical subtests, and the dependent variables were behavioral accuracy, RT, P3a amplitude and P3b amplitude, respectively. Analyses were conducted by collapsing the three tone pairs and two groups.

For the tone change detection accuracy, only the subjects’ accuracy of the out-of-key subtest significantly contributed to the accuracy data and accounted for 20.7% of the variance (p < 0.001). For the RT, again, only the out-of-key subtest reached significance, explaining 11.5% of the variance (p < 0.05). Similarly, the P3a and P3b amplitude can be significantly explained by the out-of-key subtest only, which explained 18.3% (p < 0.001) and 15.1% (p < 0.001) of the variance, respectively. The detailed results are reported in Table 1.

**Table 1:** Results of regressions analyses with the subjects’ accuracy in the three musical subtests as predictors and their tone change detection performance (accuracy and RT) and its underlying P300 activities (P3a and P3b amplitude) as predicted variables respectively.

Predictor	B	SE B	*β*	*t*	*p*
*Accuracy*					
Out of key	0.144	0.034	0.447	4.245	<0.001
Offbeat	0.041	0.039	0.115	1.061	0.290
Mistuned	−0.034	0.032	−0.107	−1.082	0.281
*RT*					
Out of key	−1.939	0.744	−0.290	−2.605	0.010
Offbeat	−0.343	0.851	−0.046	−0.403	0.687
Mistuned	−0.431	0.697	−0.065	−0.618	0.537
*P3a amplitude*					
Out of key	0.087	0.017	0.542	4.076	<0.001
Offbeat	−0.007	0.020	−0.039	−0.357	0.722
Mistuned	−0.025	0.016	−0.155	−1.551	0.123
*P3b amplitude*					
Out of key	0.066	0.015	0.483	4.435	<0.001
Offbeat	−0.004	0.017	−0.024	−0.215	0.830
Mistuned	−0.016	0.014	−0.119	−1.164	0.247

## Discussion

The current study examined the neuro-dynamic mechanism of lexical tone processing at different levels along the auditory pathway in Cantonese-speaking amusics. At the pre-attentive level, the amusics exhibited comparable MMN responses to controls, even for tone pairs with small pitch differences (T3-T6 and T2-T5). At a later, attentive level, amusics displayed overall reduced P3a amplitude for all tone pairs, and further reduced P3b amplitude for tone pairs with small pitch differences (T2-T5 and marginally significant for T3-T6). The subjects’ musical ability in the out-of-key subtest significantly predicted their accuracy and RT in detecting tonal changes and its underlying P300 activities.

Previous studies suggest that P3a and P3b are two dissociable subcomponents with different temporal and topographical distributions^41^. The P3a is associated with stimulus novelty and involuntary attentional shift to changes in the environment^41,42^; the P3b is sensitive to stimulus categorization, such that stimuli easier to categorize elicit larger P3b amplitude^41–44^. In the current study, the P3a amplitude showed an effect of pitch differences of the three tone pairs, such that the T1-T4 pair with large pitch differences elicited the largest P3a amplitude, followed by the T3-T6 pair, and finally the T2-T5 pair. This suggests easier or stronger involuntary attentional switch to more salient tonal changes. Importantly, the P3a amplitude was reduced in amusics, suggesting overall reduced attentional switch to lexical tone changes in the amusic brain. This result may be explained by reduced attentional resources in amusics; alternatively, it may reflect reduced sensitivity to pitch differences in amusics, which results in less robust automatic attentional switch to tonal changes. These two explanations are not exclusive. Interestingly, the P3a appeared to be elicited by the T1-T4 pair in the passive condition; nonetheless, no group difference was observed in the passive condition. This may suggest that different levels of attention/awareness can be revealed in ERP studies, and emphasize the importance of explicit attention requirement for disclosing the deficit of amusics.

Further group difference was observed in the P3b amplitude, which was reduced in the amusic brain for tone pairs with small pitch differences (T2-T5 and marginally significant for T3-T6) but comparable with controls for the T1-T4 pair with large pitch differences. This result suggests that the ability to actively categorize or detect lexical tone changes with small pitch differences is further impoverished in the amusic brain. Nonetheless, if the pitch differences are large, as in the case of the T1-T4 pair, the amusic brain may be able to actively categorize or detect the change. This result is consistent with previous findings that the perceptual discrimination of tone pairs with small pitch differences such as T2-T5 and T3-T6 is impaired behaviorally in amusics^23^. It is also consistent with findings on categorical perception of lexical tones that the access of phonological representations of tones is impaired in Chinese speakers with amusia^24–26^.

Overall, the results revealed that pre-attentive processing of tonal differences is normal, but the conscious detection of small lexical tone changes is impaired in Cantonese-speaking amusics. However, this finding seems to differ from the previous finding that Mandarin-speaking amusics with tone agnosia show reduced MMN responses to lexical tone changes^33^. Furthermore, previous studies have reported neural deficits in the activation of auditory cortices in the amusic brain^9,10,34^. A functional MRI (fMRI) study shows that the cerebral bases of the pitch-processing deficit in Cantonese-speaking amusics involve a broad brain network, including the right superior temporal gyrus (STG), cerebellum, right middle frontal gyrus and precuneus^11^. On the other hand, the right inferior frontal gyrus (IFG), which has been reported to exhibit deficient activation in pitch processing in a group of non-tonal language speakers with amusia^8^, appears to function normally in Cantonese-speaking amusics^11^. In light of the above finding, deficient activation of the right STG might lead to a reduction of MMN responses to lexical tone changes in Cantonese-speaking amusics. Altogether, these results imply that pre-attentive processing of lexical tone differences may be impaired.

One possible explanation for the discrepancy between the current finding and that of the previous MMN study on Mandarin tone agnosics is the severity of tone perception impairment behaviorally. In the previous MMN study, the accuracy of Mandarin-speaking tone agnosics was 3 SDs below the mean of controls in tone identification and discrimination tasks^33^. It is possible that for those amusics with most severe tone perception deficits, the impairment in the neural processing of lexical tones may be detected as early as at the pre-attentive level. A preliminary analysis was conducted in the current study to divide the 24 amusics into subgroups of tone agnosics and pure amusics. Nonetheless, the analysis failed to find differences in MMN activities between the two subgroups. All 24 amusics and 24 controls of this study participated in a post-test consisting of behavioral tone identification and discrimination tasks. Following the criteria of lower than 3 SDs in the tone discrimination task, nine amusics were determined to be tone agnosics and the remaining 15 amusics were pure amusics. However, in the identification task, even the controls’ accuracy was not very high with large SD (controls: M = 58.1%, SD = 17.5%; amusics: M = 44.9%, SD = 15.7%). As a result, only one amusics performed below 3 SDs of the mean accuracy of controls in tone identification. Therefore the subgrouping was based solely on the tone discrimination task. *Group × tone pair* ANOVAs did not find significant effects of *group* (controls vs. pure amusics vs. tone agnosics) on either the MMN latency (*F*(1, 2) = 0.176, *p* = 0.840; tone agnosics = 169 ms; pure amusics = 175 ms) or amplitude (*F*(1, 2) = 0.435, *p* = 0.650; tone agnosics = −1.235 µV; pure amusics = −1.183 µV) in the 100–250 ms time-window. There was no significant difference between tone agnosics and pure amusics in MMN latency or amplitude. Based on the preliminary results, there is no evidence that Cantonese-speaking amusics, including potential tone agnosics, demonstrated a deficit at the pre-attentive level of lexical tone processing. Future studies with a larger sample size of tone agnosics in Cantonese may further examine this issue.

The current finding also seems to differ from the finding of the previous fMRI study that the right STG shows an abnormal lack of activation during lexical tone processing in Cantonese-speaking amusics^11^. However, it should be noted that in the previous fMRI study the amusics were instructed to pay active attention to the auditory stimuli and conduct a simple task of judging whether the auditory stimuli were speech or music, whereas in the current study the amusics were instructed to ignore the auditory stimulation in the passive listening condition. It is possible that the auditory cortices may function normally or nearly normally when no focal attention was directed to the auditory stimuli. Another possibility is that different levels of awareness are better captured in ERP studies than in the previous fMRI study. This is consistent with the finding of normal activation of auditory cortices without attention to pitch deviations in amusics in another fMRI study^8^.

In light of the above discussion, the findings of the current study are consistent with previous studies on nonspeech pitch processing at pre-attentive and attentive levels^8,29,30,45^, confirming that the same neuro-dynamic mechanism presumably underlies lexical tone processing in Cantonese speakers with amusia. A domain-general neuro-dynamic mechanism thus seems to subserve the pitch-processing deficiency of amusics in music as well as speech, in that the amusic brain is able to detect small pitch differences in music and speech without awareness, but is handicapped in consciously detecting or categorizing such differences. The results are in line with the hypothesis that amusia is a disorder of awareness of pitch information^29,30,46^.

Interestingly, the “depth” of the influence of amusia along the auditory pathway seems to differ from the “depth” of the influence of musical experience. A plethora of studies have demonstrated that musicianship is associated with better pitch processing performance behaviorally, leading to smaller (better) fundamental frequency (F0) difference limen and better ability of perceptually detecting small pitch incongruities in music melodies and identifying and discriminating non-native lexical tones^47–50^. At the neural level, enhanced phonetic processing of pitch has also been reported. Without attention, larger MMN responses to nonspeech analogues of Mandarin tone contrast were found in native English-speaking musicians compared with non-musicians^51^; when active attention was required, French-speaking musicians showed an earlier peaking N2/N3 component and an enhanced P3b component to non-native lexical tone differences than non-musicians^50^.The influence of musical experience extends further up the auditory pathway to early sensory processing at the subcortical level. It has been found that the frequency-following response (FFR), auditory evoked potentials primarily generated in the brainstem in response to periodic or nearly periodic auditory stimuli, tracks pitch contour more accurately in the brain of English-speaking musicians than nonmusicans^52,53^. This demonstrates that musical experience has a “deep” impact on the auditory pathway, enhancing attentive as well as pre-attentive cortical processing of pitch, with its influence extending further up into subcortical pitch encoding. On the other hand, the influence of amusia on pitch processing along the auditory pathway appears to be “shallower”, primarily influencing later and higher level of cortical processing that involves active attention and stimulus categorization. The result of the current study together with several previous studies shows that pre-attentive processing of pitch is normal or nearly normal in the amusic brain^8,28–30^. Furthermore, subcortical FFR tracking of pitch in tonal and musical stimuli in Cantonese-speaking amusics is found to be comparable to that of controls^21^ (but see^54^ for abnormal auditory brainstem response to complex sound/da/in amusics). Having said that, the origin of the deficit along the auditory pathway in the amusic brain is an ongoing research. With more studies in the near future, especially studies examining pre-attentive and sensory processing of pitch in amusia, a fuller answer to this question can be unveiled.

To conclude, the current study found that the brain of Cantonese-speaking amusics responded to lexical tone changes normally pre-attentively, even for tone pairs with small pitch differences, but showed impairment at consciously detecting the same lexical tone changes, especially when the pitch differences were small. This finding is consistent with previous findings reported on nonspeech pitch processing. It thus provides further insights into the neuro-dynamic functioning of amusia, revealing that a domain-general neuro-dynamic mechanism may underlie the pitch-processing deficiency of amusics in the domains of music and speech.

## Methods

### Participants

Twenty-four amusics and 24 musically intact controls matched one by one in age, gender, and years of education participated in this study. All participants were native speakers of Hong Kong Cantonese. They were all right-handed, with no hearing impairment, no reported history of neurological illness or musical training. Amusics and controls were identified using the Online Identification Test of Congenital Amusia^3^. This test includes a total of three subtests, namely out-of-key, offbeat and mistuned, and has been used as a diagnostic tool for amusia in a few previous studies^6,11,22,26,55,56^. All participants took the online test in the lab under the instruction of an experimenter. Amusics scored 73% or lower, whereas controls scored 80% or higher in the global score, which was the mean accuracy of all three subtests. Furthermore, all amusics scored 71%^5^ or lower in at least one of the two pitch-based subtests (out-of-key and mistuned) or in both. Independent-samples t-tests confirmed that amusics performed significantly worse than controls in the global score (*t*(46) = −12.869, *p* < 0.001). Demographic characteristics of the participants are summarized in Table 2. Informed written consent was obtained from all participants in compliance with the experiment protocols approved by the Human Subjects Ethics Sub-committee of the Hong Kong Polytechnic University. All methods were performed in accordance with the relevant guidelines and regulations.

**Table 2:** Demographic characteristics of 24 amusic participants and 24 control participants. Amusic and control participants were determined according to the global score of the Online Identification Test of Congenital Amusia^3^ (http://www.brams.umontreal.ca/onlinetest).

	Amusics	Controls
No. of participants	24 (12 M, 12 F)	24 (12 M, 12 F)
Age (range)	22.06 ± 1.1 years	21.62 ± 1.1 years
	(20.2–24.6 years)	(19.8–24. years)
*Test of Congenital Amusia*		
Out-of-key (SD)	61.7 (10.9)	90.8 (8.3)
Offbeat (SD)	64.8 (13.9)	86.8 (7.7)
Mistuned (SD)	61.2 (13.4)	86.6 (10.7)
Global score (SD)	62.4 (8.6)	88.0 (4.6)

M = male; F = female.

### Stimuli and procedure

The stimuli were six Cantonese words contrasting six tones on the syllable /ji/: high level tone (T1) –/ji55/ ‘doctor’, high rising tone (T2) – /ji25/ ‘to lean on’, mid level tone (T3) – /ji33/ ‘meaning’, extra low level/low falling tone (T4) –/ji21/ ‘son’, low rising tone (T5) –/ji23/ ‘ear’, and low level tone (T6) –/ji22/ ‘two’. Each tone is annotated using Chao’s tone letters, which are in the range of 1–5, with 5 referring to the highest pitch and 1 referring to the lowest pitch^57^. For example, ‘55’ refers to a high level tone.

A female Cantonese speaker was recorded reading aloud these six words in isolation for ten times. For each word, one clear token was selected. All selected words were normalized to 350 ms in duration and to 75 dB in mean intensity using Praat^58^. The six tones were grouped into three pairs in forward and reversed order: T1-T4 (/ji55/-/ji21/ and /ji21/-/ji55/), T2-T5 (/ji25/-/ji23/ and /ji23/-/ji25/), and T3-T6 (/ji33/-/ji22/ and /ji22/-/ji33/). The T1-T4 pair had a large pitch difference (high level vs. extra low level/low falling tone), whereas T2-T5 and T3-T6 pairs had small pitch differences (high rising vs. low rising tone; mid level vs. low level tone). For the latter two pairs, T2 and T5 were primarily distinguished in F0 slope, whereas T3 and T6 were primarily distinguished in F0 height or mean F0. Figure 1 displays the F0 contour of the three tone pairs.

Each of the six pairs was presented in an oddball paradigm, with the first stimulus being the standard and the second stimulus being the deviant. In each block, the standard was presented frequently at a probability of 0.85, and the deviant was presented infrequently at a probability of 0.15. A total of 510 standards and 90 deviants were binaurally presented through earphones to subjects in each block. The standards and deviants were presented pseudo-randomly, such that the first eight stimuli of a block were always standards and any two adjacent deviants were separated by at least two standards. The inter-stimulus interval (offset of to onset) was 800 ms. Each block lasted about eight minutes. In total there were six blocks.

The same set of stimuli was presented twice, once in a passive listening condition, and once in an active listening condition. In the passive condition, subjects watched a self-selected muted movie with subtitles, and were instructed to ignore the auditory stimulation. The order of the six blocks was counterbalanced among the subjects as much as possible and kept identical between amusic subjects and accordingly matched control subjects. There was a break after each block. In the active condition, each block was evenly divided into two sub-blocks in order to avoid fatigue, with the probability of standards and deviants kept unchanged. This gave rise to a total of 12 sub-blocks, and each sub-block lasted about four minutes. The subjects were instructed to press a button whenever they heard a lexical tone change from the repeated standards presented at the beginning of a block. The presentation order of the 12 sub-blocks was counterbalanced as much as possible and kept identical between amusic subjects and matched control subjects. The passive condition always preceded the active condition, to avoid transfer of active attention to tonal changes in the passive condition. The two conditions were separated by at least one week for each subject.

### EEG data acquisition and analysis

EEG signals were recorded via a SynAmps 2 amplifier (NeuroScan, Charlotte, NC, U.S.A.) with a cap carrying 64 Ag/AgCI electrodes placed on the scalp at specific locations according to the extended international 10–20 system. Vertical electrooculography (EOG) was recorded using bipolar channel placed above and below the left eye, and horizontal EOG was recorded using bipolar channel placed lateral to the outer canthus of each eyes. Impedance between the reference electrode (located between Cz and CPz) and any recording electrode was kept below 5 kΩ. Alternating current signals (0.03–100 Hz) were continuously recorded and digitized with a 24-bit resolution at a sampling rate of 1000 Hz. Pre-processing of EEG signals was conducted using the BESA Version 7.1. The EEG recordings were re-filtered offline with a 0.01–30 Hz band-pass zero-phase shift digital filter (slope 12 dB/Oct in the low cutoff and slope 24 dB/Oct in the high cutoff).

For the passive condition, epochs ranging from −100 to 600 ms after the onset of each deviant and the standard immediately preceding each deviant were analyzed. Epochs with amplitudes exceeding ±75 μV at any channel were excluded from averaging. The mean acceptance rate was 74.5% (SD = 17.9%) for the amusic group and 80% (SD = 7.6%) for the control group. Independent-samples t-test found no significant group difference on the acceptance rate (*t*(31.063) = −1.361, *p* = 0.183). For each tone pair (T1-T4, T2-T5 and T3-T6), epochs were averaged across accepted trials in the two blocks where the tone pair was presented in forward and reversed order (e.g., /ji55/-/ji21/ and /ji21/-/ji55/ for T1-T4). Difference wave was obtained by subtracting the ERP waveform of the standards from that of the deviants. Following previous studies^32,38^, Fz was selected for analyzing the MMN.

Analysis was conducted on the following two time windows: MMN (100–250 ms) and late MMN or P3a (250–400 ms). As can be seen in Fig. 2, for both groups the MMN for the T2-T5 pair appeared to be delayed compared to the other two tone pairs, occurring in a late time-window of 250–400 ms. This delay is expected because the pitch divergence between T2 and T5 occurred later than the other two pairs (see Fig. 1). Also in this late time-window (250–400 ms), the T1-T4 pair appeared to elicit a P3a following the MMN, which appeared to be absent in the other two pairs. This may be due to involuntary attention shift to acoustically salient tonal changes in the T1-T4 pair^40^. *Group* × *tone pair* repeated measures ANOVAs were conducted on the MMN peak latency and mean amplitude (100–250 ms), respectively. Corrections for violations of sphericity were made using the Greenhouse-Geisser method whenever necessary. Independent-samples t-tests were conducted to analyze whether there were group differences on the late MMN for the T2-T5 pair, and on the P3a for the T1-T4 pair in the late time-window (250–400 ms).

For the active condition, epochs ranging from −100 to 800 ms after the onset of each deviant and the standard immediately preceding each deviant were analyzed. Epochs with amplitudes exceeding ±75 μV at any channel were excluded from averaging. The mean acceptance rate was 66.5% (SD = 19.7%) for the amusic group and 76.1% (SD = 17.8%) for the control group. Independent-samples t-test again revealed no significant group difference on the acceptance rate (*t*(46) = −1.767, *p* = 0.084). Furthermore, though the overall acceptance rate was lower in the active condition (71.3%) than that in the passive condition (77.2%), suggesting that the data were noisier, the components of interest, e.g., the P3a and P3b, were elicited in the active condition (see Fig. 6). For each tone pair (T1-T4, T2-T5 and T3-T6), epochs were averaged across accepted trials in the two blocks where the tone pair was presented in forward and reversed order.

Four time-windows were determined from the global field power averaged from all deviants across all electrodes (see supplementary Fig. 1) and the ERP waveforms (see Fig. 6): N1 (100–150 ms), N2b/c (250–350 ms), P3a (350–500 ms) and P3b (500–800 ms). Previous studies suggest that the N2b occurs in combination with the P3a; it is elicited by both target and non-target deviants in an oddball paradigm with a central scalp distribution^59^. The N2c often accompanies the P3b and is thought to reflect a sub-process of stimulus classification; its scalp distribution is frontocentral in the auditory modality^59^. As can be seen in Figs 6 and 7, the N2b/c was most prominent in the T2-T5 pair with a frontocentral distribution, where it appeared to accompany the P3b, which had a centro-posterior distribution. The N2b/c might reflect that more attention or cognitive control was directed to detecting tonal changes in the T2-T5 pair, where the pitch difference was small. Note that the P3a elicited in the active condition appeared to have different latency and amplitude characteristics from that elicited in the passive condition, in that the P3a in the active condition tended to occur later in time and with larger amplitude. This is consistent with the previous description that characteristics of the P300 are modulated by attention (active vs. passive)^60^. The temporal and topographical distributions of the P3a and P3b in the active condition of the current study match those reported before^41^. The selected time-windows of P3a and P3b largely overlap with those chosen in an earlier P300 study on Cantonese tone change detection^42^.

Different sets of electrodes were selected for analysis of the N1, N2b/c, P3a and P3b according to the topographic distributions (Fig. 7) and confirmed by the literature^41,42,59^. Three frontal electrodes (F3, Fz, and F4) where the N1 and N2b/c was expected to peak were selected for N1 and N2b/c analysis, and three posterior electrodes (P3, Pz, and P4) where the P300 was expected to peak were selected for P3a and P3b analysis. ERP waveforms were averaged across all selected electrodes for each tone pair. Analyses were conducted on the deviants only. The peak latency was determined from the time point with minimal (for N1, and N2b/c) or maximal deflection (for P3a and P3b) within the defined time-windows for each tone pair; the mean amplitude of each ERP component was averaged within the defined time-windows for each tone pair. *Group* × *tone pair* repeated-measures ANOVAs were conducted on the latency and amplitude of each ERP component respectively.

The behavioral accuracy and RT of the two groups of subjects in detecting tone deviants in the active condition were also analyzed. The accuracy was the percentage of deviants correctly detected for each tone pair. RT was the mean reaction time of correctly detected deviants for each tone pair. *Group* × *tone pair* repeated measures ANOVAs were conducted on the accuracy and RT respectively.

## Electronic supplementary material

Supplementary Information
