# Supplementary material for: Normal pre-attentive and impaired attentive processing of lexical tones in Cantonese-speaking congenital amusics
Source: Sci Rep. 2018 May 30;8:8420. doi: 10.1038/s41598-018-26368-7 (PMC5976652; doi:10.1038/s41598-018-26368-7)
Supplement: Supplementary file 1 — Supplementary Information [file 41598_2018_26368_MOESM1_ESM.pdf]

Normal pre-attentive and impaired attentive processing of lexical tones in Cantonese-speaking congenital amusics

Caicai Zhang[1,2,*] & **Jing Shao**[1,2]

[1]The Hong Kong Polytechnic University, Department of Chinese and Bilingual Studies, Hong Kong SAR, China
[2]Shenzhen Institutes of Advanced Technology, Chinese Academy of Sciences, Shenzhen, 518055, China
[*]caicai.zhang@polyu.edu.hk

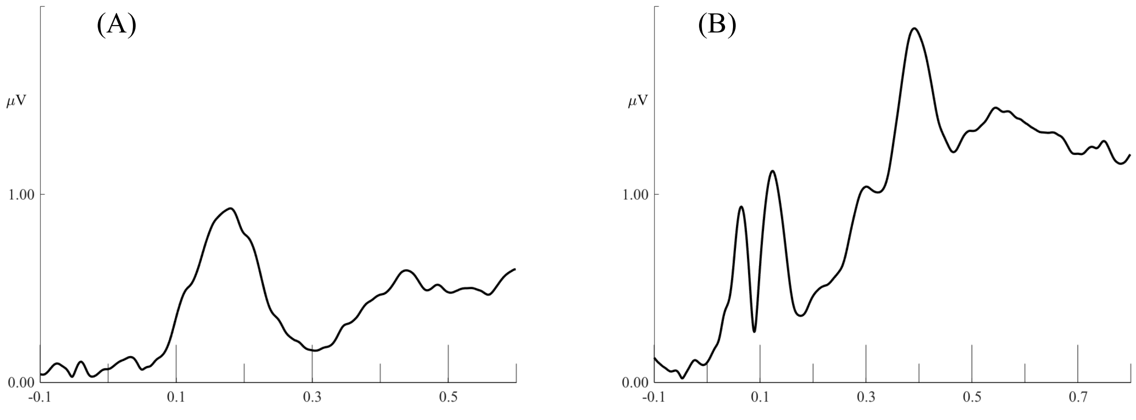
(A)
μV
1.00
0.00
-0.1
0.1
0.3
0.5
(B)
μV
1.00
0.00
-0.1
0.1
0.3
0.5
0.7
